# Exploiting a moment of weakness: male spiders escape sexual cannibalism by copulating with moulting females

**DOI:** 10.1038/srep16928

**Published:** 2015-11-26

**Authors:** Gabriele Uhl, Stefanie M. Zimmer, Dirk Renner, Jutta M. Schneider

**Affiliations:** 1 General and Systematic Zoology, Zoological Institute and Museum, University of Greifswald, Greifswald 17489; Germany; 2 Zoological Institute, Biocenter Grindel, University of Hamburg, Hamburg 20146; Germany

## Abstract

Sexual cannibalism is a particularly extreme example of conflict between the sexes, depriving the male of future reproduction. Theory predicts that sexual conflict should induce counter-adaptations in the victim. Observations of male spiders mating with moulting and hence largely immobile females suggest that this behaviour functions to circumvent female control and cannibalism. However, we lack quantitative estimates of natural frequencies and fitness consequences of these unconventional matings. To understand the importance of mating while moulting in cannibalistic mating systems, we combined mating experiments and paternity assessment in the laboratory with extensive field observations using the sexually cannibalistic orb-web spider *Argiope bruennichi.* Copulations with moulting females resulted in 97% male survival compared with only 20% in conventional matings. Mating while moulting provided similar paternity benefits compared with conventional matings. Our findings support the hypothesis that mating with moulting females evolved under sexual conflict and safely evades sexual cannibalism. Despite male benefits, natural frequencies were estimated around 44% and directly predicted by a male guarding a subadult female. Since only adult females signal their presence, the difficulty for males to locate subadult females might limit further spreading of mating with moulting females.

Sexual conflict has resulted in the evolution of many traits that control the frequency and duration of mating^1^,^2^. While traits that restrict female remating have been intensively studied^1^,^3^,^4^, pre-copulatory traits that may serve to circumvent female mating decisions are less well understood. In some species, males mate with females that are still inside the pupae or that just moulted and are still unable to move until the exoskeleton has hardened^5^,^6^,^7^,^8^,^9^,^10^,^11^,^12^. Due to the nature of such “unconventional” mating practises, it is tempting to infer sexual conflict, in that these matings are adaptive for males but in opposition to female interests.

Mating with moulting females occurs in many cannibalistic spider species^12^,^13^,^14^ and has been suggested to benefit males because females cannot move and attack males while the exoskeleton is still soft. However, sexual cannibalism that occurs during or after mating is not generally detrimental for male reproductive success, but may actually coincide with the interest of the male and lead to suicidal terminal investment in extreme cases^15^,^16^. A differentiated evaluation of the adaptive value of mating while moulting requires a comparison to conventional matings with hardened females, which is currently lacking. In the present study, we close this gap using the sexually cannibalistic and sexually size dimorphic orb-web spider *Argiope bruennichi*. This species is well suited to address this question since up to 80% of males are devoured during matings with hardened females^17^. Mating in *A. bruennichi* entails sperm transfer with the paired pedipalps, separated into two insertions each into a specific copulatory opening of the female that is connected to a separate spermatheca. Males are able to block access to a spermatheca by leaving a piece of the pedipalp behind as a mating plug^18^. Genital plugging renders the used pedipalp non-functional and limits the male to a maximum of two insertions, each with one of the pedipalps. Consequently, a male can maximally mate twice, and each time successfully plug the copulatory opening. A male that survives the first insertion can either perform his second insertion with the same female, thereby preventing her from remating with a rival male, or use his two pedipalps in two single insertions with two females.

We present a comprehensive field and experimental study that determines the frequency and conditions under which mating while moulting occurs and estimates the fitness consequences for males mating with moulting females compared to conventional mating with a fully responsive female. If *A. bruennichi* males can successfully exploit the moulting phase of the females, we predict that mating while moulting 1) reduces cannibalism, enabling the male to use each of his pedipalps and thereby achieve the maximum number of two matings, 2) increases mating duration and thus sperm transfer which should result 3) in higher relative paternity success in a sperm competitive setting.

## Results

### Assessing occurrence and reproductive success of mating while moulting under laboratory conditions

**Figure 1:**
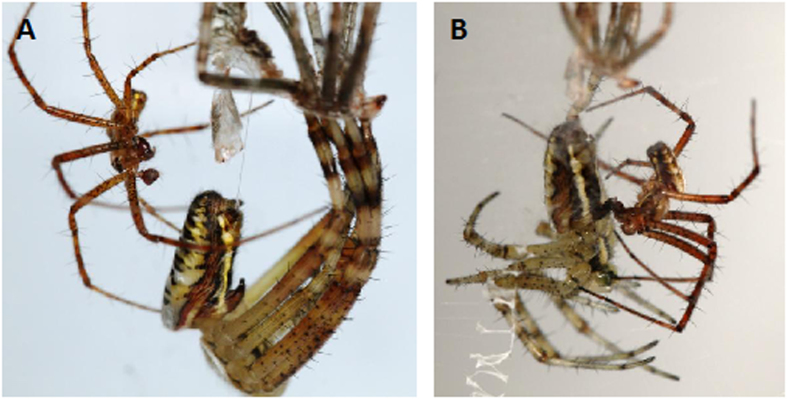
Mating while moulting in the orb-weaving spider *Argiope bruennichi*. (**A**). Female in the final phase of moulting. (**B**). Mating while moulting occurs while the female is hanging below her exuviae, still whitish (unsclerotized), and does not move.

We staged 50 male encounters with moulting females, 40 of which (80%) resulted in a successful copulation (Figure 1). Sexual cannibalism only occurred in a single case in which the male was stuck in copula, tried to uncouple without success and was consumed 38 minutes later when the female had become active.

A comparison between mating while moulting and mating with hardened females [data from^17^,^18^,^19^ revealed that first copulations with moulting females entailed a much lower risk of male death and were of a longer duration (Table 1). Only 14 of 39 surviving males (35.9%) performed a second insertion with the same female. The majority of males (25 of 39, 64.1%) did not attempt a second insertion with the same female but left the web. Twelve of 14 males (85.7%) that performed a second insertion died in copula (without female intervention) and remained attached to the female genitals until the female became active and removed them. The two surviving males had short second insertions (4 and 26 sec). The probability that male genitalia were damaged after mating^20^ was not different from matings with hardened females, nor did plugging success differ significantly^20^ (Table 1).

In order to assess whether plugging of moulting females is as effective in preventing sperm competition as in hardened females, females were remated within 24 h post-moult. 97.3% of the males (36 of 37) showed courtship behaviour and 89% of the courting males mated with the already mated female (32 of 36). Interestingly, second males were not able to detect previous usage of spermathecae despite the fact that paternity strongly depends on whether they attempt to insert into a previously used and therefore plugged or an unused spermathecae^20^,^21^. Relative paternity could be established for 31 of 32 double matings and in 28 cases we could clearly assess whether the second male inserted into a previously used copulatory duct or not. For those cases in which first and second males used different copulatory openings (N = 18), both males had equal fertilization success as in conventional matings (Table 1). In those cases in which both males used the same insemination duct (N = 10), 9 of the 10 second males gained no paternity. One male, however, mated into a used but unplugged duct and sired 65% of offspring. Hence, paternity protection and relative paternity success under competition did not significantly differ between males that mated with moulting females and males that mated with hardened females (Table 1).

### Assessing occurrence and male reproductive success of mating while moulting under natural conditions in the field

In the field, moulting took 32.85 ± 1.86 (SE) min (N = 13) on average until the female resumed her normal position in the hub of the web. Our regular patrols resulted in direct observations of moultings in 13 females of which 5 mated while moulting (38.5%). Those 5 females all had a resident male nearby before the moulting, while the other 8 did not. In 5 additional cases, we only just missed the moulting event and immediately collected the females. Using hatched offspring that these females produced in the laboratory, we determined genetically that four broods were sired by a single male and one by two males. The number of sires corresponds to the presence and number of resident males. Taken together, we observed 10 females with at least one resident male and all mated while moulting, while 8 females without a resident did not mate while moulting (X^2^ = 18.00; P < 0.001). Using presence of resident males as proxy, we can estimate the natural rate of mating while moulting to be 44%.

## Discussion

Our data demonstrate that mating with moulting females reduced the risk of cannibalism to almost zero, which is in contrast to the very high cannibalisation risk when mating with fully hardened females. Males that survive their first mating can use their second pedipalp with the same female or try to find another female for their final insertion. Our mating trials in the laboratory demonstrate that 64% of the males selected the latter, bigynous option although they could have safely mated twice with the still soft female.

These results nicely corroborate results from high risk matings with cannibalistic females where males were observed to copulate very briefly with low quality females^22^,^23^. Copulating very briefly increases survival chances from 20 to 60% and a surviving male can then leave this female and search for another, more attractive mating opportunity for his second copulation. However, since a large proportion of males do not survive their first copulation it remains unclear how often *A. bruennichi* females prevent males from realising their optimal mating investment during conventional mating interactions. In this study, male mating decisions are revealed uninfluenced by female behaviour due to the absence of an aggressive response.

Recent literature challenges the long held notion that male mate choice will only evolve under rare circumstances and requires paternal investment. Indeed, the now propagated view is that male mate choice can evolve in diverse mating systems characterised by a high male breeding cost^24^. Our study system is a good example for high mating costs due to sexual cannibalism and the limitation to two copulations due to genital damage while males do not provide paternal investment. Hence, the existence of male mate choice in *A. bruennichi* provides evidence that high mating effort alone can select for male mate choice^25^,^26^.

Males that mate with moulting females do not only benefit from surviving the copulation, but achieve much longer insertion durations. In matings with hardened females, longer copulation duration is positively correlated with the amount of sperm being transferred into the spermatheca before plugging it^17^. In a sperm competition setting in which a subsequent male uses the second spermatheca, relative copulation duration predicts relative fertilization success^17^. However, the relatively longer first copulations of males that mated with moulting females did not translate into a higher paternity share. Several mutually non-exclusive explanations for this finding are conceivable. Firstly, the soft exoskeleton of a moulting female may cause mechanical difficulties in sperm transfer or disadvantageous sperm storage conditions because the spermathecae may not be prepared yet for hosting sperm^27^. Secondly, females may cryptically select against males mating with them during the moult e.g. through differentially storing sperm or differentially activating sperm from different spermathecae^28^,^29^,^30^. In both scenarios, an advantage in controlling access to mating partners does not translate into an advantage in sperm competition. There are examples from several taxa in which post-copulatory sexual selection does not amplify pre-copulatory processes^31^,^32^,^33^.

In the laboratory, where males were placed on webs of subadult females, 80% mated with the moulting female. In the field, mating while moulting was exclusively performed by guarding males and only 44% of subadult females had a resident male. Pre-copulatory guarding is favoured under conditions of first male sperm precedence and if mating is restricted in time^34^ and both conditions apply to *A. bruennichi*. The question arises why not more males guard immature females in order to achieve a low risk mating while the female moults to maturity. One explanation for why guarding and mating while moulting is not more common might be that cohabiting with an immature female entails opportunity costs for the male. Waiting until the female moults may take days and thereby valuable time is lost^35^. The magnitude of these costs will depend on concurrent availability of alternative mating options. Indeed, in our northern study populations in which *A. bruennichi* females mature relatively synchronously, opportunity costs are likely to arise for males that guard females that are not among the first to mature.

A second important factor is the costs of finding an immature female. For example, in *Heliconius* butterflies males are known to find female pupae by cueing in on host plants of larvae^36^. However, female pupae also produce a volatile pheromone that helps males to find and guard them, suggesting that females benefit from being found and guarded^37^. In *A. bruennichi*, males cannot detect subadult and very young adult females from a distance because the male-attracting pheromone is only produced by hardened females^38^ and males do not seem to be able to exploit a moulting cue. Accordingly, encounters between males and subadult females likely occur rather haphazardly if roving males come across silk of subadult females. The chance of encountering a web will likely be a function of female density. Density effects that determine mate encounter rates are generally known to shape mating strategies and mating systems^39^. Our model system would be ideal to explore the interaction between density and synchrony on male mating strategies further since these spiders show variation in population dynamics across geographic and environmental gradients^40^. Proximate questions that remain to be investigated are how males identify a conspecific female and how they decide on staying or leaving.

Mating while moulting clearly prevents the female from exerting control over mating frequency and duration and females may be monopolized by a male against their interest, constituting sexual conflict. Selection on female counter-measures to regain control will be a function of the relative costs of losing control, which will depend on the variance in male quality, on mate encounter rate i.e. population density^41^, and the probability of receiving a mating while moulting. In a hardened state, females generally accept every male for a single copulation but exclude the majority of males from future matings through sexual cannibalism. This leaves one sperm storage organ available for another male that may be of higher quality than the first one. However, the average mating rate of females in the field was found to be only 1.3^42^ and polyandry per se does not produce obvious benefits for the female^29^ with the exception of the reduction of inbreeding costs^43^,^44^. In concordance with our findings, a recent meta-analysis demonstrated that potential benefits of polyandry may indeed be small when no direct benefits are involved^45^. Females may in fact profit from mating while moulting if we assume costs of signalling. The volatile pheromone that hardened females emit is highly effective in attracting males, but since it is a derivative of a primary metabolite, it may have to be traded off against basic metabolism^38^,^46^. However, even without metabolic costs, chemical signalling may provoke indirect costs through predation and costly excess of males^47^. Females likely benefit from saving any expenditure into mate attraction and signalling without compromising fertilization. Females that mate while moulting can immediately start investing resources into egg-production without the risk of having to deposit an egg-sac that is not fertilized. Depending on the balance between the above mentioned benefits and the negative consequences that arise from lack of mate choice, the net-costs of mating while moulting may indeed be modest for females.

In summary, mating with a moulting female can be considered a male response to sexual cannibalism in *A. bruennichi.* Our study shows that this mating strategy is established in natural populations. The highly increased survival probability in the first copulation enables males to opt for their maximal mating rate of two and to exert mate choice. Whether this male strategy selects for female counter-adaptations depends on the benefits of female mate choice and polyandry relative to its costs. It is noteworthy that in mating systems with low male mating rates as in sexually cannibalistic systems, male mate choice will evolve and harmonise the interests of both sexes approaching a situation of life-long monogamy and complete overlap of interests. Under such conditions females may not suffer as strongly from losing control as in species with conventional sex roles leading to co-occurrence of alternative mating behaviours.

## Methods

### Laboratory study

100 juvenile and subadult females and 120 subadult males were collected from meadows near Greifswald, Germany, (54° 6'20.14″N, 13°20'8.81″E) at the beginning of July 2011 and were reared in individual containers. Rearing conditions including feeding regime was chosen similar to those of a previous study on mating behaviour with hardened *A. bruennichi* females^19^. Courtship and mating behaviour in matings with hardened females were described in previous studies^17^,^19^. When in the subadult stage and close to the final moult, 50 females were checked for moulting activity every 20 minutes. When a moulting event was due, which can be assessed by the posture of the female in her web, a male was carefully introduced to the periphery of the web. A few seconds later, we added a eunuch male to the web, whose copulatory organs, the pedipalps, had been removed under anaesthesia. Eunuchs were used to simulate a male-male competition scenario as it was suspected to occur in the field and for ease of comparison with previous mating experiments that were performed under similar conditions^13^,^19^. Male and female behaviours were recorded throughout the observation period of 45 min from introducing the males. After the focal male had performed one pedipalp insertion, he was observed for another 10 minutes to see if he continued with a second insertion using the other pedipalp. In matings with hardened females, males that escape female cannibalistic attacks (with appr. 20% probability^17^) either use their second pedipalp within this time frame or leave the web of the female. After mating, the focal male as well as the rival male was removed. Within 24 h a second fertile male and another eunuch were introduced to the female. Mating behaviour of second focal male was also observed for 45 minutes and if matings occurred, we again registered the duration of copulation, whether the male conducted one or two pedipalp insertions and the occurrence of male death during copulation.

After the mating trials, males were preserved in 70% ethanol and later inspected for genital damage. Pedipalps were scrutinized for the loss of the whole sperm transferring sclerite, the embolus, or parts of it^18^. As these broken-off pieces function as mating plugs only if they physically block the genital opening of the female^18^,^20^, we also inspected the two insemination ducts of each female for the presence of plugs. After oviposition, female genitalia were dissecting out and the soft tissue was dissolved in a 5% solution of NaOH. In this transparent state, stuck emboli tips can be detected inside the female copulatory ducts^18^.

We used the irradiated male technique to assess relative paternity success of two successive mating partners^48^. Therefore, half of the males were irradiated with a dosage of 40 Gray (Siemens MEVATRON) at the radiology department of the University Clinic of Greifswald. This procedure is well established and ideal for a laboratory setting with few mating partners^49^. Two experimental double mating groups were used: first male irradiated and second male non-irradiated and vice versa. Eggs fertilized by sperm from the irradiated male do not develop. Consequently, unhatched eggs can be attributed to the irradiated male and hatched eggs to the non-irradiated male. A correction factor for natural failure of development was applied^17^.

Our data from mating trials with moulting females were compared with data from previous studies with hardened females: mating behaviour in the presence of a eunuch rival^19^; genital damage and plugging probability^18^; paternity success^17^.

### Field study

The field study took place on a meadow in Lower Saxony, Germany (53°06'14.83″N, 11°47'20.64″E) from 08.07.2011 until 19.07.2011. The study site was open grassland. Shortly before the mating season we marked 50 web locations of subadult females with a bamboo stick along an established path. Females that are approaching their final moult to adulthood are clearly distinguishable since their genital region (epigyne) protrudes from the body surface like in adult females but is still covered by a cuticular layer.

During daily patrols (2–3 times a day), we noted the number of males in and around (ca. 50 cm radius) the webs of subadult females (termed resident males). After the first three females had been found moulted, we started a continuous patrolling scheme along the path, resulting in six inspections per hour per subadult female. To allow individual recognition and night observations, 23 subadult females that seemed close enough to their final moult were individually marked with non-toxic paint (KREMER Pigmente, Aichstetten, Germany) on their opisthosoma while in their webs. In addition, we noted their identity on poles next to their webs. When a female started to moult to maturity we documented the duration of the moulting process, the behaviour of male residents, whether new males arrived at the web and particularly whether mating while moulting occurred. We then registered the number of mating partners and the occurrence of sexual cannibalism. Individual continuous observation ended when the female became active, i.e. moved into the hub of her web from where she typically hunts. During the first five days we inspected the focal females routinely over 24 h. Since it turned out that moulting events did not occur at night, the patrolling was done from 6 am to 11 pm. The moulting process of another five females could not be observed since they moulted while another female was observed at the same time. However, moulting events of these females were registered as soon as the patrolling routine was resumed. In order to assess whether these females had mated unobserved and if so, with how many males, we collected these females for genetic analyses and transferred them to the laboratory at the University of Hamburg. They were kept in individual 500 ml plastic cups, sprayed with water five days a week and fed with three *Calliphora* spec. on two days a week. The egg sacs of the females were incubated and opened four weeks after oviposition. Females and her offspring were preserved at −80 °C for genetic analyses. Paternity was assessed for 20–40 offspring per female using 16 polymorphic microsatellite markers^50^. Determination of paternity was done by direct comparison of the identified alleles between offspring and mother.

We extracted DNA from the spiderlings and their mothers with the 5 PRIME ArchivePure DNA Kit according to the manufacturer’s protocol (5 PRIME, Hamburg, Germany). We used whole spiderlings for DNA extraction and half of the amount of extraction solution recommended in the ArchivePure DNA Kit protocol. PCR amplification was performed according to the Qiagen Multiplex PCR Kit Protocol (Qiagen, Hilden, Germany). Genotyping was performed on an Applied Biosystems 3730 DNA Analyzer with ABI ROX size standard at the MPI for Evolutionary Ecology in Plön, Germany. Microsatellite alleles were called using GeneMapper 4.0 (Applied Biosystems).

### Statistics

Data were analysed with the statistical program JMP 10 and SPSS 21. Descriptive statistics are given as mean ± standard error (SE) or median (interquartile range, abbreviated as IQR). Sample sizes may differ between analyses due to missing data.

## Additional Information

**How to cite this article**: Uhl, G. *et al.* Exploiting a moment of weakness: male spiders escape sexual cannibalism by copulating with moulting females. *Sci. Rep.* **5**, 16928; doi: 10.1038/srep16928 (2015).

## Figures and Tables

**Table 1:** Comparison of mating parameters between laboratory matings with moulting females (this study) and with hardened, cannibalistic females from previous studies^17^,^18^,^19^.

Parameters	Moulting females	Hardened females	Statistics
Duration of 1. insertion in s	13.00 (9.10) N = 40	7.00 (3.25) N = 34^a^	U = 260.50 **p < 0.001**
Cannibalism after	1:39 (2.5%)*	26:8 (76.5%)^a^	X^2^ = 43.39 **p < 0.001**
1. insertion (yes:no)			
Pedipalp mutilated after	37:3 (92.5%)	15:1 (93.8%)^a^	X^2^ = 0.03 p = 0.870
1. insertion (yes:no)			
Plugging success after	32:6 (84.2%)	107:12 (90%)^b^	X^2^ = 0.92 p = 0.337
1. insertion (yes:no)			
P_2_ - used opening	0.00 (0.00) N = 10	0.54 (0.57) (N = 19)^a^	U = 44.50 p = 0.119
P_2_ - unused opening	0.50 (0.28) N = 18	0.00 (0.09) (N = 13)^a^	U = 153.50 p = 0.559

Data on copulation duration pertain to the first pedipalp insertion, since survival probability of males that mate with hardened females is only about 20%^17^,^19^. Pedipalp mutilated: loss of the sperm transferring sclerite, the embolus. Plugging success: probability with which the embolus can be found stuck in the female copulatory duct. P_2_: paternity of the second male. Data are given as medians (interquartile range) or as occurrences (proportions).

^*^male stuck in copula, tried to free himself without success, cannibalized after 2100 sec by female that had hardened in the meantime.

^a^data taken from reference^19^.

^b^data taken from reference^18^.
